# Nutrition education intervention for dependent patients: protocol of a randomized controlled trial

**DOI:** 10.1186/1471-2458-12-373

**Published:** 2012-05-24

**Authors:** Victoria Arija, Núria Martín, Teresa Canela, Carme Anguera, Ana I Castelao, Montserrat García-Barco, Antoni García-Campo, Ana I González-Bravo, Carme Lucena, Teresa Martínez, Silvia Fernández-Barrés, Roser Pedret, Waleska Badia, Josep Basora

**Affiliations:** 1 Atención Primaria. Institut Català de la Salut, Tarragona, Spain; 2 Institut d’Investigació en Atenció Primària IDIAP Jordi Gol, Catalunya, Spain; 3 IISPV, Universitat Rovira i Virgili, Tarragona, Spain; 4 Centre d’Atenció Primària Sant Pere, Camí de Riudoms, 53-55, 43202 Reus, Tarragona, Spain

## Abstract

**Background:**

Malnutrition in dependent patients has a high prevalence and can influence the prognosis associated with diverse pathologic processes, decrease quality of life, and increase morbidity-mortality and hospital admissions.

The aim of the study is to assess the effect of an educational intervention for caregivers on the nutritional status of dependent patients at risk of malnutrition.

**Methods/Design:**

Intervention study with control group, randomly allocated, of 200 patients of the Home Care Program carried out in 8 Primary Care Centers (Spain). These patients are dependent and at risk of malnutrition, older than 65, and have caregivers. The socioeconomic and educational characteristics of the patient and the caregiver are recorded. On a schedule of 0–6–12 months, patients are evaluated as follows: Mini Nutritional Assessment (MNA), food intake, dentures, degree of dependency (*Barthel test*), cognitive state (*Pfeiffer test*), mood status (*Yesavage test*), and anthropometric and serum parameters of nutritional status: albumin, prealbumin, transferrin, haemoglobin, lymphocyte count, iron, and ferritin.

Prior to the intervention, the educational procedure and the design of educational material are standardized among nurses. The nurses conduct an initial session for caregivers and then monitor the education impact at home every month (4 visits) up to 6 months. The North American Nursing Diagnosis Association (NANDA) methodology will be used. The investigators will study the effect of the intervention with caregivers on the patient’s nutritional status using the MNA test, diet, anthropometry, and biochemical parameters.

Bivariate normal test statistics and multivariate models will be created to adjust the effect of the intervention.

The SPSS/PC program will be used for statistical analysis.

**Discussion:**

The nutritional status of dependent patients has been little studied. This study allows us to know nutritional risk from different points of view: diet, anthropometry and biochemistry in dependent patients at nutritional risk and to assess the effect of a nutritional education intervention. The design with random allocation, inclusion of all patients, validated methods, caregivers’ education and standardization between nurses allows us to obtain valuable information about nutritional status and prevention.

**Trial Registration number:**

Clinical Trial Registration-URL: http://www.clinicaltrials.gov. Unique identifier: NCT01360775

## Background

There is evidence that malnutrition is common in the elderly and may influence the prognosis associated with several pathological processes, loss of independence, decrease of quality of life, and increase of morbidity-mortality and hospital admissions [1-3].

Malnutrition prevalence has been reported as 3–5% in free-living older adults and 11.6% to 60% in institutionalized individuals [4,5] but a recent study showed 21.3% in home care patients [5].

Educational programs for the elderly, including nutritional advice, have observed improvement in health status [6]. In Barcelona, a controlled trial was conducted in chronically ill 65-year-old patients and healthy controls. The intervention consisted of a self-care program as well as education on physical activity, nutrition, and social support; a statistically significant difference was observed in nutritional status [7].

Aging is related to a loss of functional capacity, and the role of caregiver becomes crucial to quality of life and for both prevention and treatment of malnutrition in dependent patients. A caregiver support program for hospitalized dependent patients observed that the support obtained positive results for the dependent patients [8]. In Finland, a nutrition education program based on constructive learning theory was developed to educate professional caregivers of patients with dementia. After one year, the study concluded that education had positive effects on the nutrition of patients [9].

However, educational interventions for caregivers of Alzheimer patients in France [10] and Parkinson patients in Europe [11] have not proven effective.

The Mini Nutritional Assessment Test (MNA), commonly used to assess nutritional status because it can be done quickly, has been validated for screening and assessment of malnutrition in older people, including the use of a reduced version [12-14]. Harris et al. noted that the MNA test has a sensitivity of 80% and a specificity of 90% [15]. Different programs have affirmed the ability of the geriatric nutritional risk index to assess the nutritional status of patients at home [16,17]. A study using the MNA found that 67.6% of subjects treated in a Home Care Program and 93.1% of the institutionalized subjects were malnourished or at risk of malnutrition; with adjusted data, this risk was 4.4 times higher among Home Care Program patients than institutionalized ones [18].

While the use of MNA as a screening tool is well established, the assessment of nutritional status and in particular of the changes produced by an intervention should have more specific estimator instruments. A recent study observes that age, sex, and body mass index (BMI) are responsible for 11.3% of the variability of the MNA test, whereas MNA items related to diet are responsible for 62.5% of this variability, indicating the importance of diet on the change in nutritional status. On one hand, this observation indicates that the most important items affecting improvement of nutritional status are the adequacy of the patient’s diet and, on the other, that more accurate methods of assessing food consumption are needed to determine the change in the diet [19].

Furthermore, the most important risk factors for malnutrition in elderly patients have been identified as number of teeth [20], depression [21], and dementia [22]. This indicates the importance of assessing these health problems in addition to the MNA test, and of this entire assessment being done by nurses in patient care programs at the household level [23].

A Home Care Program for dependent patients has been developed by Primary Health Care (PHC) services in our environment to guarantee continuity of care, access to services, and equality in care of these patients who for various reasons cannot go to a Primary Care Center (PCC).

The aim of the study is to assess the effect of a nutritional education intervention aimed at caregivers on the nutritional status of dependent patients at risk of malnutrition.

## Methods

### Design

The study design is a randomized controlled trial; intervention consists of nutritional education for caregivers of approximately 200 dependent patients at risk of malnutrition, conducted in the Home Care program in various PCC of the Tarragona-Reus area.

**Figure 1:**
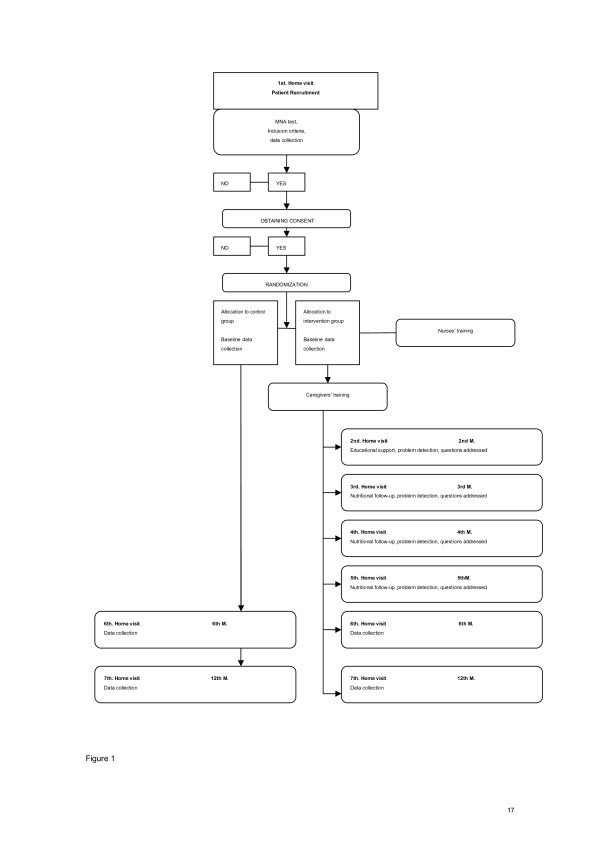
Research procedure.

Research procedure is diagrammed in Figure 1.

### Study population

Subjects will meet the following inclusion criteria: 1) participation in the Home Care program (ATDOM), 2) aged 65 years or older, 3) MNA score from 17 to 23.5 points, and 4) must have a caregiver. Exclusion criteria are: 1) MNA score outside the range of 17 to 23.5 points, 2) enteral feeding required, 3) severe dysphagia, 4) any serious illness that progresses to malnutrition, and 5) consumption of vitamin and/or dietary supplements.

### Recruitment

Random selection of 8 PCCs of the Catalan Institute of Health (ICS) in Reus and Tarragona counties, stratified to represent different geographical areas: a) 4 PCCs in 2 cities over 100,000 population (2 in Reus and 2 in Tarragona), b) 2 PCCs in the suburbs of these cities (1 in Tarragona, 1 in Reus), c) 1 PCC in a medium-sized urban area (about 30,000 inhabitants), and d) 1 PCC in rural areas.

Subjects will be recruited by initial identification in the electronic medical record (e-cap) indicating that they meet the criteria for participation, to be verified by performing a baseline MNA test.

### Randomization of groups

After subjects provide signed written informed consent, they will be classified randomly. From a common database, subjects will be computer-assigned to the intervention group and non-intervention (control) group in each PCC, proportionally to the total number of subjects. Subjects who choose not to participate in the study will be replaced by others of the same sex, age, and PCC to obtain the initial total sample of 97 subjects in each group.

### Intervention

Educational session aimed at caregivers: A one-hour, standardized educational session, conducted by a nurse in small groups of 15 caregivers will have the following content:

• Nutritional value of food.

• Designing a healthy diet.

• Advice on dietary adaptation to address the most common nutritional problems in this group, such as energy, protein, vitamin, mineral and water deficiency, and adaptation of textures.

• Recommendations on basic cooking techniques.

Monitoring of educational intervention: PCC nurses will monitor patients monthly at home up to 6 months and then at 12 months.

To provide individualized dietary advice as necessary, standardized ad hoc cards have been developed with all the predictable interventions.

Controls: Control subjects will not receive nutritional intervention; they will be visited once to complete an initial assessment and will receive regular Home Care follow-up at 6 months and 12 months.

### Training

Four 2-hour sessions will be held to standardize the procedure for nurses. Briefings are intended to standardize the procedure and train the nurses of the different PCC participants.

Content of the first briefing includes:

• Presentation of the study.

• Data collection procedures: MNA, Food Frequency Questionnaire (FFQ), Barthel, Yesavage and Pfeiffer questionnaires.

The second session will focus on the educational intervention:

• Reminder of the general concepts of nutrition and food in nutritional risk situations.

• Methodology for nutrition education aimed at the elderly dependent’s caregiver.

The third session will be aimed to train nurses for dietary assessment and detection of critical points. The objectives to achieve during the month will be set from these critical points.

The fourth session will have the following content:

• Standardization of education for caregivers.

• Creation of educational material.

• Procedures.

These sessions will be conducted by expert nutrition researchers with experience in educating health professionals.

### Outcomes Assessment

Subject variables in the intervention and non-intervention groups: These variables will be collected at baseline and except sociodemographic variables and current medical history again at 6 months (end of intervention), and 12 months. For the intervention group there is a questionnaire on adherence to the diet.

### Primary outcomes measures

Nutritional status variables: *Key variable:* MNA (Mini Nutritional Assessment) is a validated test with sensitivity and specificity for the diagnosis of malnutrition [12]. MNA has been selected as the main variable because it is the most used tool in PHC.

#### Other variables

##### Anthropometric measurements

Determination of standing height: Height will be measured at the highest point of the head, barefoot, with knees together, not bent, feet together, head up and eyes forward.

Calculation of length at knee-to-heel height (in the case of bedridden persons): With the person supine, flex the knee to an angle of 90. The measure will require 2 people: one will fix solid references at level of heel and knee, and the other will measure it with a tape measure. To estimate height, the formula of Chumlea will be used [24]:

$$\begin{array}{l} \text{Man}:\left(2.02\text{ × knee height }–\text{ cm heel}\right)–\left(0.04\text{ × age years}\right)+64.19\\ \text{Woman}:\left(1.83\text{ × knee height }–\text{ cm heel}\right)–\left(0.24\text{ × age years}\right)+84.88 \end{array}$$

Determination of body weight: Portable scale. Subject will be standing in light clothes and barefoot, with the patient at the center of the scale.

Determination of body weight when it cannot be measured: the following formula will be applied:

$$\begin{array}{l} \text{Man}:\left(\text{MUAC × }2.31\right)+(\text{CC × }1.5)–\;50.1\\ \text{Woman}:\left(\text{MUAC × }2.31\right)+(\text{CC × }1.43)–\;37.46 \end{array}$$

Middle-Upper Arm circumference (MUAC): we will use the midpoint of the arm. With arm relaxed and parallel to the body, we will determine the circumference at this point with tape, without tightening the tape [14].

Calf circumference (CC): we will follow similar instructions.

*Daily consumption of food* Food frequency questionnaire (FFQ): Knowledge of the dietary intake of individuals is a fundamental tool to assess the risk of disease. This test is validated for the population of Reus and gives information on food intake, energy, and micronutrients [25].

*Biochemical markers* The biochemical parameters to be analyzed are:

• Serum albumin and prealbumin (chemiluminescence).

• Serum transferrin (immunoturbidimetric).

• Hemoglobin, hematocrit, lymphocyte count (Coulter).

• Serum iron (colorimetric using ferrozine as chromatogenous agent).

A unique study profile will be created to analyze all these parameters in the Tarraco laboratory (ISO 9001:2000 certified ICS Tarragona laboratory). The extraction will be done in patient homes by PHC nurses. The same professional will take it to the PCC in regular temperature conditions, using the standard sample transport procedure between PHC and the central laboratory. The samples will be stored 8 days, the usual period of storage in the laboratory, and the results will be sent to the PHC within 8 days.

#### Secondary outcome measures

##### Sociodemographic variables

Age and sex will be recorded and social risk will be assessed through assessment of the family socio-scale designed and validated by Primary Health Care of Gijón [26].

##### Current medical history

Chronic diseases registered (e-cap), type of teeth (natural or dentures, and if prosthesis, good or bad fit).

##### Degree of dependency

Barthel Test or Basic Activities of Daily Living (ADL), used to make a physical functional assessment of patients. The collection is done by direct observation or by asking the patient if possible. The total score is calculated by adding the score of each activity. A higher score indicates greater independence: High dependence (<30), moderate dependence (30–60) and mild dependence (60–90) [27].

##### Cognitive function

Assessment of cognitive impairment by Pfeiffer’s test, a validated screening questionnaire of cognitive impairment, is fast and allows corrections depending on the subject’s level of education [28].

##### Mood

Rating mood of the population 65 years old and over by Yesavage Depression Scale identifies the presence of depression and sets levels. It consists of a series of questions with the responses scored as 0 or 1 point. A 5-item version will be used in this study. Scores are classified as 0–1, no depression; ≥ 2, possible depression [29].

### Caregiver variables in the intervention and non-intervention groups

These variables will be collected at baseline for both groups, except for the assessment of knowledge acquisition, which will be collected in the intervention group before the intervention, immediately after, and at 1–6–12 months thereafter.

#### Secondary outcome measures

##### Sociodemographic data

Age, sex, socioeconomic status, education, and the patient’s connections (family, volunteers, employee).

##### Knowledge acquisition

An 11-item questionnaire on basic concepts explained in the nutritional education intervention, designed by researchers.

### Statistical analysis

#### Sample size

The main variable is the MNA score. This value has an approximate average of 25.4 and standard deviation of 3.7 points in the Spanish population [15]. It is considered that variation of 2 points is a clinically relevant value. Considering a one-sided hypothesis and admitting an alpha error of 0.05 and a power of 95%, a sample of 72 subjects in each group is needed to observe a difference of 2 points in the MNA test after educational intervention with a precision of 80%. Taking into account possible losses during follow-up, the sample expands by 20% (15 subjects) so that the study includes 87 subjects in each group, a total of approximately 200 patients.

#### Analysis strategy

Variables will be described as mean and standard deviation and percentages for quantitative and qualitative variables respectively. Comparison of quantitative variables will use Student-Fisher *T* test and analysis of variance for categorical variables and the chi-square statistical test. Standard tests (Kolmogorov-Smirnov and Shapiro-Wilks) will be used to verify the hypothesis of normal distribution of quantitative variables. If the preconditions for the application of statistical tests are fulfilled non-parametric tests will be used.

Longitudinal studies with a control group, which act in a phased manner on the caregiver-patient to see the effect on the patient, have different directions of analysis: caregiver assessment to measure acquisition of knowledge and implementation of dietary advice, and patient assessment to measure the effectiveness of educational intervention on nutritional status (MNA, diet, anthropometry, biochemistry) at 6 and 12 months in the intervention and control group and between groups.

To evaluate these effects, multivariate models of multiple linear regression and logistic regression will be created.

To measure the effect of educational intervention on the patient’s nutritional status (MNA, diet, anthropometry, biochemistry), the results will be adjusted according to degree of knowledge acquisition and implementation of dietary advice, as well as other variables that may interfere with the patient’s nutritional status such as socio-economic characteristics, caregiver education levels, and patients’ chronic diseases, dental status, degree of dependence (Barthel test), cognitive function (Pfeiffer Test), or mood (Yesavage Scale).

Initially the inclusion in the model of all variables that are part of the theoretical model will be forced, and in a second phase there will be an automatic selection by the stepwise method (backward and forward) to get reduced models.

The conditions of application of the models will be verified by standard techniques that are primarily based on analysis of the residuals.

The bilateral null hypothesis of normality, no difference and no significance of regression coefficients, will be discarded when their probability is less than 5%.

For data analysis the latest version of the software package SPSS/PC for Windows will be used.

### Ethical Approval

The study protocol was approved by the Ethics Committee of the Institute of Primary Care Research (IDIAP) Jordi Gol on April 27, 2009.

## Discussion

Conducting a randomized intervention study allows us to compare the effect of an educational intervention about nutritional aspects between two groups, intervention and control, and random geographic location of the subjects avoids bias.

Inclusion of the PCCs of our area is randomized and stratified representing different geographical areas according to various categories: rural, urban, city center and suburb. The inclusion in the study of all patients in the Home Care program of the selected PCCs is impor-tant to avoid selection bias.

Educational intervention is aimed to caregivers because its efficacy in improving the health of dependent patients has been observed. Inclusion of the educational intervention in a Home Care Program already being carried out by nurses facilitates the conduct of the study and guarantees continuity if positive results are obtained.

The same assessment methodology will be used for the intervention and non-intervention groups and different nurses will conduct the groups to avoid influence between groups in the majority of the centers, except in the smallest centers where the same nurse will necessarily conduct both groups.

Education will be standardized among the nurses and will use the North American Nursing Diagnosis Association (NANDA) methodology specific to the nursing profession. Nursing diagnosis is the clinical judgment about an individual response to life processes, including real or potential health problems, and provides the basis for the choice of nursing intervention in order to reach the goals or outcomes. These results should be formulated before identifying and performing interventions, which are those actions that are aimed at helping the patient to achieve the expected results based on knowledge and clinical judgment [30].

Inclusion of NANDA methodology will improve the effect of early education of caregivers because it continues nutritional counselling in a direct and personalized way, nurses will use their own professional methodology, and this one is internationally accepted.

Study results will be obtained for the main variable MNA test, a validated screening test with a sensitivity of 80% and a specificity of 90% [14], and also using other outcomes measures like anthropometric measurements (estimated according to standard recommendations), a validated food frequency questionnaire, and biochemical markers. Standard laboratory methodology will be followed for extraction, transport, treatment and storage of the samples, which will be frozen for simultaneous analysis to avoid analytical variability.

Losses to follow-up are a limitation of longitudinal studies. Moreover, in dependent patients, the loss is very high due to their serious health condition. The 20% increase over the minimum required sample and the effect at 6 months will be calculated for intermediate reporting in the case of subjects lost to follow-up.

## Competing interests

The authors declare that there is no conflict of interest.

## Authors' contribution

All authors provided comment, read and approved the final version of the manuscript. Study concept and design: VA, NM, TC, CA, AIC, MGB, AGC, AIGB, CL, TM. Conducted research: VA, NM, TCA, RP, WB, JB, Coordinators for data collection: VA, NM, TC. Aquisition of data: TC, CA, AIC, MGB, AGC, AIGB, CL, TM. Planning of statistical analysis, database design: VA, NM, SFB: Drafting of the manuscript and primary responsibility for final content: VA, SFB. All authors read and approved the final manuscript.

## Pre-publication history

The pre-publication history for this paper can be accessed here:

http://www.biomedcentral.com/1471-2458/12/373/prepub
